# Adaptive Aquila Optimizer Combining Niche Thought with Dispersed Chaotic Swarm

**DOI:** 10.3390/s23020755

**Published:** 2023-01-09

**Authors:** Yue Zhang, Xiping Xu, Ning Zhang, Kailin Zhang, Weida Dong, Xiaoyan Li

**Affiliations:** School of Opto-Electronic Engineering, Changchun University of Science and Technology, Changchun 130022, China

**Keywords:** Aquila Optimizer (AO), chaotic distribution, adaptive adjustment, Niche, global optimization, swarm intelligence

## Abstract

The Aquila Optimizer (AO) is a new bio-inspired meta-heuristic algorithm inspired by Aquila’s hunting behavior. Adaptive Aquila Optimizer Combining Niche Thought with Dispersed Chaotic Swarm (NCAAO) is proposed to address the problem that although the Aquila Optimizer (AO) has a strong global exploration capability, it has an insufficient local exploitation capability and a slow convergence rate. First, to improve the diversity of populations in the algorithm and the uniformity of distribution in the search space, DLCS chaotic mapping is used to generate the initial populations so that the algorithm is in a better exploration state. Then, to improve the search accuracy of the algorithm, an adaptive adjustment strategy of de-searching preferences is proposed. The exploration and development phases of the NCAAO algorithm are effectively balanced by changing the search threshold and introducing the position weight parameter to adaptively adjust the search process. Finally, the idea of small habitats is effectively used to promote the exchange of information between groups and accelerate the rapid convergence of groups to the optimal solution. To verify the optimization performance of the NCAAO algorithm, the improved algorithm was tested on 15 standard benchmark functions, the Wilcoxon rank sum test, and engineering optimization problems to test the optimization-seeking ability of the improved algorithm. The experimental results show that the NCAAO algorithm has better search performance and faster convergence speed compared with other intelligent algorithms.

## 1. Introduction

With the continuous development of science and technology, the effective integration of life sciences and engineering sciences has become the main feature of modern science and technology [1], and meta-heuristic algorithms have flourished under this trend. Currently, optimization objectives in practical engineering applications range from single objective to multi-objective, from continuous to discrete, and from constrained to unconstrained, with complex problems. The drawbacks of traditional optimization algorithms are obviously due to their heavy reliance on initial values and their tendency to fall into local optima. Meta-heuristic optimization algorithms are easy to implement, bypass local optima, are non-derivative mechanisms, and do not require gradient information [2,3,4,5]. Compared with the two types of algorithms, the performance of meta-heuristic algorithms is outstanding and has received widespread attention from scholars; they have not only played an important role in the field of computing, but have also shown powerful problem-solving capabilities in the fields of military [6], agriculture [7] and hydraulic engineering [8].

Since the series of algorithms such as Particle Swarm Optimization [9] (PSO), Genetic Algorithm [10] (GA) and Artificial Bee Colony [11] (ABC) have been proposed, they are continuously applied in many engineering examples and provide better solutions to the problems [12]. In the process of rapid development, scholars mainly focus on population initialization, population exploration and how to quickly converge to the global optimum to study meta-heuristic algorithms in depth. Abualigah L et al. proposed a new meta heuristic algorithm in 2021: Aquila Optimizer (AO) [13]. The algorithm, inspired by the prey-catching behavior of the Aquila, has demonstrated in the generalized arithmetic test that AO has a high performance and high local optimum capability in the exploration and development stages and in the process of handling composite functions compared to 11 algorithms such as GOA, EO, DA, PSO, ALO, GWO, and SSA. However, its local exploitation capability is insufficient and easy to fall into a local optimum and slow convergence, and many scholars have improved it. Wang S et al. [14] combined the development phase of Harris Hawks Optimization (HHO) with the exploration phase of AO to propose an improved hybrid Aquila Optimizer and Harris Hawks Optimization (IHAOHHO), combining both nonlinear escape energy parameters and stochastic opposition-based learning strategies to enhance the exploration and development of algorithms in standard test functions and industrial engineering design problems, demonstrating strong superior performance and good promise. Verma M et al. [15] generated a population by a standard AO and a new population by a single-stage genetic algorithm based on the concept of evolution, where binary tournament selection, roulette wheel selection, shuffle crossover and replacement mutations occur to generate a new population. The chaotic mapping criterion is then applied to obtain various variants of the standard AO technique. The standard AO is further improved, yields better results and is applied to engineering design problems. However, the effect of the homogeneity of chaotic systems on population initialization is not considered. Akyol S [16] used the tangent search algorithm with an intensive phase (TSA) to optimize the AO and proposes the Skyhawk Optimizer tangent search algorithm (AO-TSA). The enhancement phase (TSA) of the tangent search algorithm is used instead of the limited exploration phase to improve the exploitation capability of the Aquila Optimizer (AO), while the local minimum escape phase of the TSA is applied in the AO-TSA to avoid the local minimum stagnation problem. Mahajan S et al. [17] proposed a new hybrid approach using Aquila Optimizer (AO) and Arithmetic Optimization Algorithm (AOA) by combining AO with Arithmetic Optimization Algorithm (AOA). Efficient search is achieved in high- and low-dimensional problems, further showing that the population-based approach achieves efficient search results in high-dimensional problems. Zhang Y J et al. [18] similarly combined AO with Arithmetic Optimization Algorithm (AOA) and proposed The Hybrid Algorithm of Arithmetic Optimization Algorithm with Aquila Optimizer (AOAAO). An Individual exploration and development process in the energy parameter E equilibrium population is also introduced, and segmented linear mappings are introduced to reduce the randomness of the energy parameters. The proposed AOAAO is experimentally validated to have a faster convergence speed and higher convergence accuracy.

In addition, AIRassas A M et al. developed an improved Adaptive Neuro-Fuzzy Inference System (ANFIS) via AO: AO-ANFIS [19] for predicting oil production and experimentally demonstrated that AO significantly improved the prediction accuracy of ANFIS. Abd Elaziz M et al. [20] proposed a new image of COVID-19 classification framework using DL and a hybrid swarm-based algorithm that use AO as a feature selector to reduce the dimensionality of the image. The experimental results showed that the performance metrics of feature extraction and selection stages are more accurate than other methods. Jnr E O N et al. [21] proposed a new DWT-PSR-AOA-BPNN model for wind speed prediction and for efficient grid operation by combining the Skyhawk Optimization Algorithm (AOA) with Discrete Wavelet Transform (DWT), Phase Space Reconfiguration (PSR) with Chaos Theory, and Back Propagation Neural Network (BPNN). Meanwhile, AO has been applied to population prediction [22], PID parameter optimization [23], power generation allocation [24], path planning [25] and other fields. Due to the short time of AO proposal, its slow convergence speed and its tendency to fall into local optimum still need further optimization and improvement. It is important to further improve its performance and make it applicable to more practical problems.

The literature shows that these algorithms can effectively approach the true expectation of a multi-objective problem. The problem of zero search preferences, however, has not attracted much attention from scholars. The effective solution of this problem is the basis for improving the robustness of the AO algorithm and allows the algorithm to be applied to new classes of problems or to propose new optimization algorithms. This is the purpose and significance of this work, in which we propose a new algorithm called Adaptive Aquila Optimizer Combining Niche Thought with Dispersed Chaotic Swarm (NCAAO), based on the recently proposed Aquila optimizer (AO). The research gaps and contributions of the paper can be summarized as follows:

- The NCAAO has great potential in solving various engineering problems such as feature selection, image segmentation, image enhancement and engineering design.
- The AO and NCAAO have rarely been combined with other available meta-heuristics; therefore, the NCAAO has a great potential to be combined with other algorithms available in the literature.
- Hence, a new chaotic mapping is introduced which has better uniformity, better initializes the population, and effectively enhances the efficiency of the algorithm.
- This new algorithm is further investigated in terms of search thresholds, which allows the population to adaptively adjust the search space by changing the search threshold. The introduced adaptive weight parameter perturbs the population of individual positions and improves the algorithm local exploitation ability.
- Moreover, for the algorithm to obtain the optimal solution with high accuracy, a communication exchange strategy based on the idea of Niche thought is proposed to ensure the optimization-seeking accuracy and convergence speed of the algorithm by screening elite individuals.

In summary, in order to solve the problem that the convergence of AO is slow and easy to fall into local optimum, firstly and as inspired by the literature [26], the method of initializing population is adopted to improve the robustness of the algorithm. The Aquila population is initialized by DLCS chaotic mapping. The approach we have used in this study aims to enhance the homogeneity of the Aquila population in the search space and improve the randomness and ergodicity of the population individuals. Second, an adaptive adjustment search strategy for de-searching preferences is proposed to balance global exploitation and local search by the adaptive threshold adjustment method. On this basis, the adaptive location weighting method is introduced to perturb the update of the group individuals’ location and improve the local exploitation ability. The de-search preference adaptive adjustment search strategy can remove the search preference problem effectively to a certain extent and help the algorithm escape from the local optimum quickly. Finally, inspired by literature [27] and combined with the elite solution mechanism, a communication exchange strategy based on the idea of Niche thought is used to ensure that the goodness of the population is maintained during the iterative process. Through the information exchange among individuals, the global optimum is better selected and the rapid convergence of the scout individuals to the global optimum is promoted to enhance the robustness of the algorithm.

The rest of the paper is structured as follows: Section 2 describes the principle of AO and studies its module performance. Section 3 describes the proposed algorithm and analyzes the time complexity of the proposed algorithm. Section 4 verifies the robustness and applicability of the algorithm through numerical experiments and engineering experiments. Section 5 summarizes the full text and examines the future research direction.

## 2. Aquila Optimizer

### 2.1. Mathematical Model of AO

The AO algorithm simulates the different hunting styles of Aquila for different prey. For fast-moving prey, the Aquila needs to acquire the prey in a fast and precise way, by which the global exploration ability of the algorithm is reflected. Similarly, the local exploitation ability of the algorithm is reflected by the hunting way for slow-moving prey. The optimization process is represented by simulating four types of Aquila hunting behaviors.

First, the population needs to be generated randomly between the upper bound (UB) and lower bound (LB), specified based on the problem, as shown in Equation (1). The approximate optimal solution during the iterative process up to each iteration is determined as the optimal solution. The current set of candidate solutions *X* is randomly generated by Equation (2).

(1) $$X=\left[\begin{array}{ccc} x_{1,1} & \cdots & x_{1,D}\\ \vdots & \ddots & \vdots \\ x_{n,1} & \cdots & x_{n,D} \end{array}\right]$$

(2) $$X_{i,j}=rand\times (UB_{j}-LB_{j})+LB_{j},i=1,2,\ldots ,N\;j=1,2,\ldots D$$

where *n* denotes the total number of candidate solutions, *D* denotes the dimensionality of the problem and *x_n,D_* denotes the position of the nth solution in the *D*th dimensional space. Rand is a random number, *UB_j_* denotes the *j*th dimensional upper bound of the given problem and *LB_j_* denotes the *j*th dimensional lower bound of the given problem.

#### 2.1.1. Expanded Exploration(X_1_)

The first way is to select the search space by flying high in a vertical bend. Aquila flies high to recognize prey areas and quickly select the best prey area. This behavior is shown by Equation (3).

(3) $$X_{1}(t+1)=X_{best}(t)\times (1-\frac{t}{T})+(X_{M}(t)-X_{best}(t))\times rand$$

(4) $$\;X_{M}(t)=\frac{1}{N}\sum_{i=1}^{N}X_{i}(t),\forall j=1,2,\ldots ,D$$

where *X*_1_(*t* + 1) denotes the position of an individual at time *t* + 1, *X_best_*(*t* + 1) denotes the current global best position at the *t*th iteration, *T* and *t* denote the maximum number of iterations and the current number of iterations, respectively, *X_M_*(*t*) denotes the average position of an individual in the current iteration, and *Rand* is a random number between 0 and 1 in the Gaussian distribution.

#### 2.1.2. Narrowing the Scope of Exploration (X_2_)

The second approach is short gliding attacks in isometric flight. Aquilas hover above the target prey in preparation for an attack when they spot the prey area from a high altitude. This behavior is represented by Equation (5).

(5) $$X_{2}(t+1)=X_{best}(t)\times levy(D)+X_{R}(t)+(y-x)\times rand$$

(6) $$levy(D)=s\times \frac{u\times \sigma }{{\left|v\right|}^{\frac{1}{\beta }}}$$

(7) $$\sigma =\left(\frac{\Gamma (1+\beta )\times \sin(\frac{\pi \beta }{2})}{\Gamma (\frac{1+\beta }{2})\times \beta \times 2^{\left(\frac{\beta -1}{2}\right)}}\right)$$

where *X*_2_(*t*+1) is the generated solution for the next iteration of t, *D* denotes the spatial dimension, *levy(D)* is the Lévy flight distribution function, X_R_(*t*) is the random position of the Aquila in [1,N], *s* takes the value of 1.5, and *y* and *x* present a spiral situation in the search space as shown in the following equation:

(8) $$y=r\times \cos\left(\theta \right)$$

(9) $$x=r\times \sin\left(\theta \right)$$

(10) $$r=r_{1}+0.00565\times D_{1}$$

(11) $$\theta =-0.005\times D_{1}+\frac{3\times \pi }{2}$$

where *r*_1_ takes a fixed index between 1 and 20, and *D*_1_ is an integer from 1 to the length of the search space.

#### 2.1.3. Expanded Development (X_3_)

The third way is a low-flying and slow-descent attack. The Aquila locks onto a hunting target in the hunting area, and with the attack ready, makes an initial attack in a vertical descent, thus further testing the prey’s response. This behavior is represented by Equation (12).

(12) $$X_{3}(t+1)=(X_{best}(t)-X_{M}(t))\times \alpha -rand+((UB-LB)\times rand+LB)\times \delta$$

where *X*_3_(*t* + 1) is the solution of the next iteration of t generated, *δ* and *α* are both mining adjustment parameters in the range of (0,1), and UB and LB represent the upper and lower bounds of the given problem.

#### 2.1.4. Narrowing the Development Area(X_4_)

The fourth way is walking and grabbing prey. When the Aquila approaches the prey, it attacks the prey according to the random movement of the prey. This behavior is represented by Equation (13).

(13) $$X_{4}(t+1)=QF\times X_{best}(t)-(G_{1}\times X(t)\times rand)-G_{2}\times levy(D)$$

(14) $$QF(t)=t^{\frac{2\times rand-1}{{\left(1-T\right)}^{2}}}$$

(15) $$G_{1}=2\times rand-1$$

(16) $$G_{1}=2\times (1-\frac{t}{T})$$

where *X*_4_(*t* + 1) is the generated solution for the next iteration of t; *QF* denotes the mass function used to balance the search strategy and $F\in \left(0,1\right)$; *G_1_* denotes the different methods used by the Aquila for prey escape; *G_2_* represents the slope value from the first position to the last position during the Aquila’s prey chase, taking a decreasing value from 2 to 0; *Rand* is a random number between 0 and 1 in the Gaussian distribution; and *T* and *t* denote the maximum number of iterations and the current number of iterations, respectively.

### 2.2. AO module Performance Split Study

In order to extract the modules with more prominent optimization performance, the standard AO modules are separated and tested. The other modules are masked in the code source, one module is selected to update the individual positions, and the typical arithmetic cases F1, F10, and F13 in the literature [5] are selected to run the AO after the separation optimization process for ten times; the data are shown in Table 1, where *F_fit* is the average fitness of the ten times.

**Figure 1:**
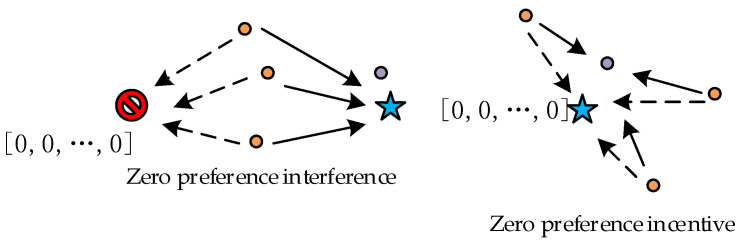
Zero preference interference and incentive.

Two phases in the AO algorithm introduce a greedy algorithm to perturb the individual positions to ensure that the new position of the individual is better than the original position. The four positions’ update formulas are observed by the module separation test described above. In Equation (3), when the optimal solution is 0, it converges to 0. When the optimal solution is not 0, it converges to a constant; in Equation (5), it barely converges to the optimal solution; and in Equations (12) and (13), it mostly converges to a constant. Through the analysis of Equation (3), it is found that the optimal value of 0 creates an incentive for the algorithm to converge to the optimal solution quickly during the iterative process. Therefore, the algorithm easily falls into the local optimum, and the probability that the local optimum is 0 is larger, which will be called the zero-point search preference. The zero-point preference interference and incentive schematic are shown in Figure 1. There is a zero-point preference corollary as follows.

There exist *N* feasible solution vectors in each dimension in the search domain $\subset {\left[LB,UB\right]}_{D}$ defined by the function *F(x)*; then, there is a total of *N_D_* feasible solution vectors in the search domain. If there exist *K* (*K* is a constant) solutions of *F(x)* with the same global optimal fitness and *N* tends to infinity, the probability that any feasible solution vector in the search domain corresponds to a global optimal fitness solution is as in Equation (17).

(17) $$P\{fitness(X)=\min(fitness)\}=\underset{N\to \infty }{\lim}\frac{K}{N^{D}}$$

If the algorithm has no preference, the search probability for each feasible solution should be of the same order as *P{fitness(X) = min(fitness)}*. Preference appears when a feasible solution vector *X^p^* in the search domain is attractive to the population, then there is Equation (18).

(18) $$P\{fitness(X)=\min(fitness)\}=\underset{N\to \infty }{\lim}\frac{K}{N^{D}}$$

Find the fitness probability of the global optimum, and then determine whether there is interference in the search process; if there is no interference, then it is expressed by Equation (19).

(19) $$P\{fitness(X_{best})\}<\underset{N\to \infty }{\lim}\frac{K}{N^{D}}$$

Therefore, it can be judged that the probability of the population converging to *X_best_* tends to be a higher order infinitesimal.

## 3. Improved Algorithm

### 3.1. Generation of Group Based on Chaotic System

The goodness of the initial population directly affects the solution accuracy and convergence speed of the algorithm, and a well-diversified initial population can largely improve the performance of the algorithm. The problem of using a stochastic system to generate the initial population in optimization problems is that there is a lack of correlation between previous and upcoming states; so, the upcoming states cannot be predicted, resulting in an uneven distribution of the initialized population.

Compared with random systems, chaotic systems are more sensitive to initial values and have the characteristics of mixing properties, randomness, ergodicity and regularity [28,29,30], which can produce uniform and stable orbits. Given a long enough time, a chaotic system can traverse all states of the space, and the traversal process never repeats in a certain range, so it can effectively circumvent the local optimum. Lin Z B and Liu Y H [31] proposed the DLCS chaotic mapping by adding a piecewise model to the Lorenz chaotic mapping and introducing a greedy strategy. The DLCS chaotic mapping has extremely complex kinematic properties with outstanding initial value sensitivity, high distribution uniformity and stability, and an enhanced search uniformity of the algorithm for neighborhood solutions. The expressions are shown in Equations (20)–(23), and *Z_sn+1_* is the main chaotic sequence generated.

(20) $$X_{n+1}=X_{n}+a\cdot (X_{n}-Y_{n})\cdot \Delta s$$

(21) $$Y_{n+1}=Y_{n}+(b\cdot X_{n}-X_{n}\cdot Z_{n}-Y_{n})\cdot \Delta s$$

(22) $$Z_{n+1}=Z_{n}+(X_{n}^{2}+\sin(X_{n}\cdot Y_{n})-c\cdot Z_{n})\cdot \Delta s$$

(23) $$Zs_{n+1}=Z_{{}_{n+1}}\cdot 16\mathrm{mod}Gear$$

where a = 10, b = 28, c = 6.2 and Δs is the step size taken as 0.01; the signal amplifier control parameter is taken as 16, and Gear = 105 for the sequence slice mode parameter.

**Figure 2:**
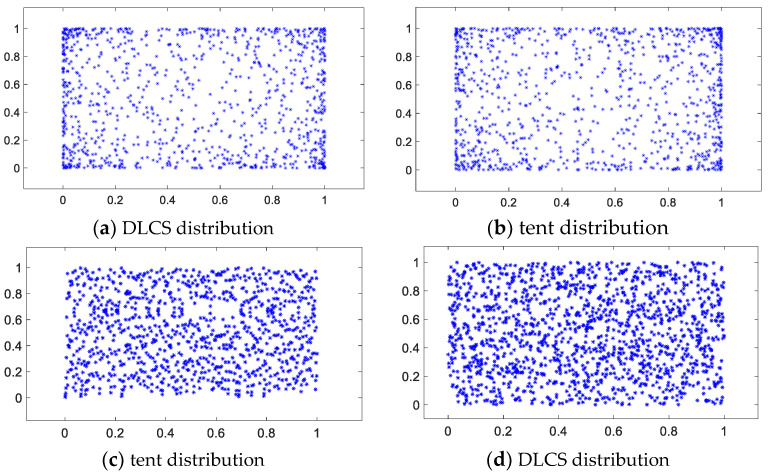
Chaotic System Planar Distribution.

The logistic mapping [32], cubic mapping [33], tent mapping [34] and DLCS mapping were used to generate sequences of a length of 1000 each on initial values with small differences and normalized to form a two-dimensional planar distribution in the interval [0, 1] for 30 uniformity comparison experiments; the metrics that constitute the chaotic model are shown in Table 2. When the ratio of both adjacent statistical values of a chaotic distribution graph is closer to 1, the uniformity of the chaotic system is higher. To observe the uniformity of the chaotic orbits generated by the two chaotic systems, two perturbed orbits were generated using the four chaotic systems mentioned above to form a chaotic distribution map, as shown in Figure 2. It is obvious that logistic and cubic have an edge over the density phenomenon, which leads to a weaker ergodicity of the chaotic system. Although most of the segments of the tent map converge normally, there are still uneven distributions. Combined with Table 2, it can be seen that DLCS has the relatively best performance with a stable and uniform distribution, which is suitable for generating the initial chaotic repulsive group of individuals.

### 3.2. Adaptive Adjustment Strategy for De-Searching Preferences

The fitness in the AO algorithm reflects the difference between the individual and global optimum. In the AO algorithm, it is shown how to quickly choose a search strategy that causes the Aquila to reach the area where the prey is located in the fastest way and thus obtain the prey (optimal solution). The adaptive adjustment strategy of the de-searching preference uses two methods: the adaptive probability threshold method and the adaptive position weight method. First, to further improve the convergence speed of AO while balancing the ability of global exploration and local exploitation, an adaptive probability threshold method is proposed. In this method, the adaptive probability threshold adaptively changes between 0 and 1 with the iterations of the algorithm, guiding individuals to quickly select the hunting strategy more suitable for the current population at different times. The mathematical expression of the adaptive probability threshold *adaptive_p* is shown in Equation (24).

(24) $$adaptive\_p=1-\left[\frac{1}{1+\lambda }\times \left(\lambda \times \frac{t^{\lambda }}{T^{\lambda }}+\mu \times \frac{t^{\mu }}{T^{\mu }}\right)\right]$$

where *λ* and *μ* are control parameters and take the values *λ* = 3, *μ* = 2, and *T* and *t* denote the maximum number of iterations and the current number of iterations, respectively.

Therefore, the adaptive probability threshold is introduced into the Gaussian distributed random number *Rand* to further and precisely select the appropriate strategy for hunting. The mathematical expression for the hunting mode of AO can therefore be re-expressed as Equation (25).

(25) $$X(t+1)=\left\{\begin{array}{c} X_{best}(t)\times (1-\frac{t}{T})+(X_{M}(t)-X_{best}(t))\times rand\\ X_{best}(t)\times levy(D)+X_{R}(t)+(y-x)\times rand\\ (X_{best}(t)-X_{M}(t))\times \alpha -rand+((UB-LB)\times rand+LB)\times \delta \\ QF\times X_{best}(t)-(G_{1}\times X(t)\times rand)-G_{2}\times levy(D) \end{array}\begin{array}{c} ,ifrand<adaptive\_p\\ ,ifrand\geq adaptive\_p \end{array}\right.$$

At the beginning of the algorithm iteration, the adaptive probability threshold is larger, guiding the Aquila population for global exploration. At the later stage of algorithm iteration, the adaptive probability threshold is smaller to guide the populations for local exploration. This enables the algorithm to adaptively adjust the search strategy and reduce the search preference while effectively improving the convergence speed of the algorithm.

Second, the adaptive weight method is introduced to adjust the position update of AO. The adaptive weight coefficients are shown in Equation (26).

(26) $$\xi =\frac{1}{\tau +\mu }\times \left(\tau \times \frac{t^{\tau }}{T^{\tau }}+\mu \times \frac{t^{\mu }}{T^{\mu }}\right)$$

where τ = 2, μ = 2 and $\xi \in \left[0,1\right]$.

When ξ increases with the number of iterations, it indicates that the prey selected after each iteration is also the optimal solution for the current population with its own adaptation degree, which produces a stronger attraction to the Aquila in the population. At the same time, the credibility of the information conveyed by the randomly selected individuals in the population increases under the iteration, thus making the Aquila able to find the prey more accurately according to the adaptive weight change, thus improving the algorithm convergence speed and the ability to find the best. However, when the local exploitation is carried out in the late iteration of the algorithm, the Aquila approaches the prey; a relatively small weight coefficient should be used at this time so that the individuals update their position while finely searching whether there is a more optimal solution around the prey, thus improving the local exploitation capability of the algorithm. The introduction of adaptive weight coefficients to update the position is shown in Equations (27)–(30).

(27) $$X_{1}(t+1)=\xi \times X_{best}(t)\times (1-\frac{t}{T})+(X_{M}(t)-X_{best}(t))\times rand$$

(28) $$X_{2}(t+1)=\xi \times X_{best}(t)\times levy(D)+X_{R}(t)+(y-x)\times rand$$

(29) $$X_{3}(t+1)=((1-\xi )\times X_{best}(t)-X_{M}(t))\times \alpha -rand+((UB-LB)\times rand+LB)\times \delta$$

(30) $$X_{4}(t+1)=QF\times (1-\xi )\times X_{best}(t)-(G_{1}\times X(t)\times rand)-G_{2}\times levy(D)$$

### 3.3. Communication Strategy Based on Niche Thought

**Figure 3:**
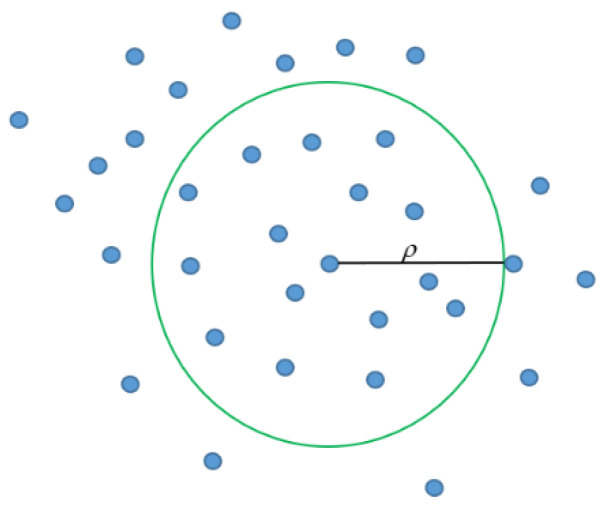
Diagram of Niche Thought.

Niche thought comes from biology, where microhabitat refers to the functions or roles of organizations in a particular environment, and organizations with common characteristics are called species. Niche can help maintain diversity in the biological community and help form new species in nature. A schematic diagram of Niche thought is shown in Figure 3.

Niche thought is introduced in the AO algorithm [35], which uses a sharing mechanism to compare the distance between individuals in a habitat. A specific threshold is set to increase the fitness of individuals with high fitness, which is used to ensure that the individual state is at the optimum. For individuals with low fitness, a penalty is given to make them update and further search for optimal values in other regions to ensure the diversity of the population in the iterative process and further obtain the location of the optimal solution. In this process, the distance between the individuals of the small habitat population is calculated by Equation (31).

(31) $$d_{ij}=\left|X_{i}-X_{j}\right|$$

The information exchange function between individual Xi and Xj is shown in Equation (32).

(32) $$sh(d_{ij})=\left\{\begin{array}{c} 1-\frac{d_{ij}}{\rho },d_{ij}<\rho \\ 0,\;d_{ij}>\rho \end{array}\right.$$

where ρ is the radius of information sharing in the microhabitat, and $d_{ij}<\rho$ ensures that individuals survive in the microhabitat environment.

After sharing information, the optimal adaptation is adjusted in time, as shown in Equation (33).

(33) $$F_{i}\_best=\frac{F_{i}}{sh},i=1,2,\ldots ,N$$

where $F_{i}\_best$ is the optimal adaptation after sharing, and $F_{i}$ is the original adaptation.

### 3.4. Algorithm General Framework

**Figure 4:**
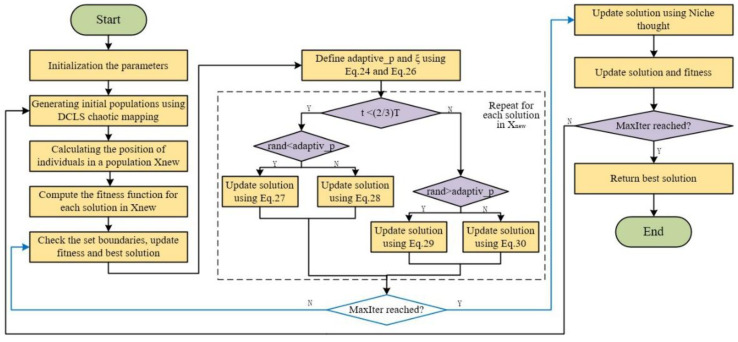
NCAAO algorithm flow chart.

In summary, the improved modules are reorganized to constitute the new algorithm after the improved AO, which is called the Adaptive Aquila optimizer Combining Niche Thought with Dispersed Chaotic Swarm(NCAAO). The overall flow chart of the algorithm is shown in Figure 4. In NCAAO, a set of candidate solutions is generated by chaotic mapping to initiate the improved algorithm. Through repeated iterations, the search strategy of NCAAO gradually converges toward the optimal solution or obtains the location of the optimal solution. The adaptive adjustment strategy and the communication exchange strategy help the algorithm to obtain the optimal position according to the optimization process, balancing global exploration with local exploitation. Until the algorithm reaches the end criteria, the NCAAO search process is terminated. The Algorithm 1 pseudo-code is as follows.
**Algorithm 1. Pseudo-code of NCAAO**Initialization phase:Initialization of the parameters of the AO (i.e., D, UB, LB, t, T)Generating initial populations using DCLS chaotic mapping with a length ofChaoticTrackLength_no and a range of [LB, UB]_D_ X_i_ = (i = 1,2,...0., N)**while** (t < T + 1)Calculate the fitness function values.  X_best_(t) = Determine the best obtained solution according to the fitness values.Update x, y, G_1_, G_2_, Levy(D), etc.Update adaptive_p and ξ using Equations (24) and (26)**For** i = 1:N^D^**if rand<adaptive_p**  Update the position of X(t + 1) using Equation (27)  **Else**
  Update the position of X(t + 1) using Equation (28)   **End if**
**Else****if rand>adaptive_p**  Update the position of X(t + 1) using Equation (29)  **Else**
  Update the position of X(t + 1) using Equation (30)   **End if**
Calculate distance from X_i_ to X_j_ using Equation (31)Update the best fitness using Equation (33)**End for****End**

### 3.5. Time Complexity Analysis

The computational complexity of the improved algorithm is based on the original algorithm by adding chaotic mapping to generate the initial population and updating the fitness of the population through Niche thought. The computational complexity of the algorithm still depends on the initialization of the solution, the calculation of the function and the update of the solution. First, if the number of individuals in the initialized population is N, the dimension of the objective function is D and the maximum number of iterations is T. The time complexity is expressed in terms of “*O*”, and the time complexity of solving the initial population of Aquila once per solution is Equation (34).

(34) O(T×D)

The process of updating the solutions includes updating the positions of all solutions as well as exploring the best positions with a time complexity of Equation (35).

(35) $$O\left(N\times T)+O(N\times T\times D\right)$$

Through the small habitat idea to ensure the excellence of the population in the iterative process and repeated iterations to compare the mutual distance between the population particles and adjust the optimal adaptation in time, the time complexity is as in Equation (36).

(36) O((N−1)×T×D)

Therefore, the total algorithm time complexity is shown in Equation (37). Since the chaotic orbit length and the number of iterations need to be determined based on the solution problem, the actual complexity of the algorithm is influenced by the actual problem.

(37) O(D×(N×T))

## 4. Algorithm Performance Experiments

In this section, the performance of the proposed NCAAO in solving optimization problems is studied. For this purpose, fifteen objective functions of NCAAO were selected from the literature [36,37] for solving different types of unimodal, high-dimensional multimodal and fixed-dimensional multimodal modes. The benchmark functions used are detailed in Table 3. Similarly, three engineering design problems were selected to test the applicability of NCAAO.

In addition, the obtained optimization results from the proposed NCAAO are compared with five well-known optimization algorithms. These competing algorithms include popular methods: Gray Wolf Optimization (GWO) [38], Whale Optimization Algorithm(WOA) [39], Harris Hawks Optimization(HHO) [40], Ant Lion Optimizer(ALO) [41] and Aquila Optimizer(AO) [13]. Table 4 shows the values of the control parameters of these algorithms.

To evaluate the performance of optimization algorithms, each of the competing algorithms as well as the proposed NCAAO in 30 independent implementations (with each independent implementation containing 500 iterations) have been implemented on the objective functions. The experimental environment is Windows 10, a 64-bit operating system, the CPU is Inter Core i7-10750H, the main frequency is 2.60 GHz and the memory is 16GB. The algorithm is based on MATLAB R2019a. The simulation results are reported using two criteria: the average of the best solutions obtained (AVG) and the standard deviation of the best solutions obtained (STD).

### 4.1. Benchmark Set and Compared Algorithms

**Figure 5:**
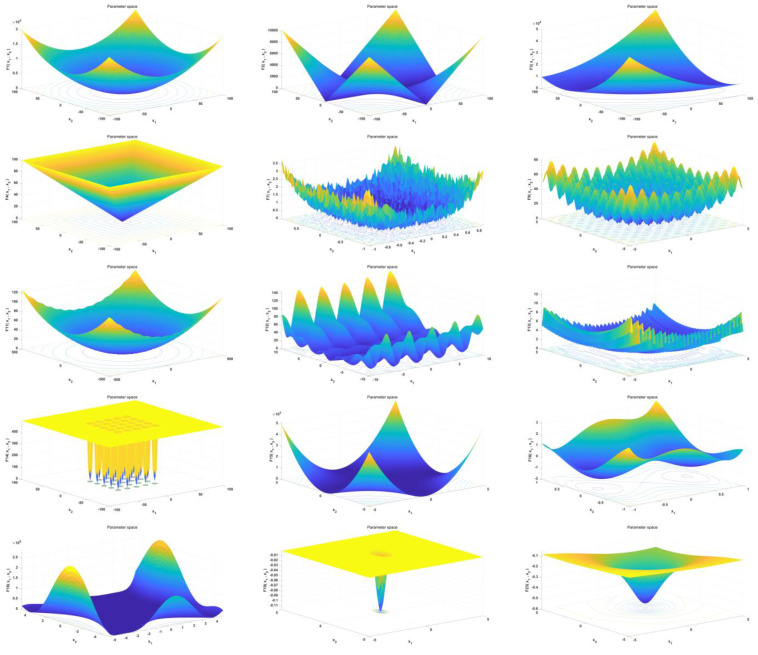
Fitness charts of 15 cases in two dimensions.

Table 3 includes five unimodal functions (F1–F5) to test the algorithm development capability, four high-dimensional multimodal functions (F6–F9) to test the algorithm search capability as well as the local optimum avoidance capability, and six fixed-dimensional multimodal modes (F10-F15) that are considered to be a combination of the first two sets of random rotations, shifts and biases. The composite test functions are more similar to the real search space, facilitating the simultaneous benchmarking of algorithm exploration and development [42]. The ‘w/t/l’ in Table 3 indicates the number of wins, draws and failures of each algorithm. The algorithms in Table 3 are shown in Figure 5 in the graph of the effect of adaptation in two dimensions.

The NCAAO algorithm was tested by the unimodal functions, and the results are shown in Table 5. As can be seen from the table, the NCAAO algorithm is able to provide very competitive results on the single-peak test function, especially on the F2 function for all dimensions with significant improvements. The proposed algorithm converges to the global optimum of this function, i.e., zero. This proves that the proposed algorithm has a high utilization capacity, which is due to the role of initializing the population with the DLCS chaotic map, helping the NCAAO algorithm to provide high utilization. Therefore, the NCAAO algorithm has a strong development capability.

After proving the exploitation of NCAAO, we will discuss the exploration of this algorithm. The NCAAO algorithm was tested by high-dimensional multimodal functions, and the results are shown in Table 6. From the table, it can be seen that the NCAAO algorithm is equally competitive with the classical algorithm in finding the optimal values for different dimensions of the F6 and F7 functions. However, the results of finding the optimum for F8 and F9 functions show that the NCAAO algorithm has a stronger search ability and local optimum avoidance ability than other classical algorithms. The excellent exploration of the proposed algorithm is due to the adjustment of adaptive search thresholds and the introduction of adaptive position weighting parameters. Directing a population from one search region into another promotes exploration. This is similar to the crossover operator in GA that highly emphasizes search space exploration.

F10–F15 are the six objective functions that evaluate the ability of the optimization algorithm to handle fixed-dimensional multimodal mode problems. The results of optimizing these objective functions using the proposed NCAAO and the competing algorithms are shown in Table 7. The proposed NCAAO optimizing F10, F12, F14, and F15 is able to converge to the global optimum of these functions. NCAAO is the first best optimizer for solving F14 and F15. In optimizing the functions of F10, F11, F12 and F13, although the average criterion of NCAAO is similar to some rival algorithms, its standard criterion is more outstanding. Therefore, the proposed NCAAO solves these objective functions more effectively. The analysis of the simulation results shows that the proposed NCAAO has a higher ability to solve the fixed-dimensional multimodal mode optimization problems from F10 to F15 compared with six competing algorithms.

Although the use of AVG and STD indices from the experimental evaluation showed that the NCAAO algorithm outperformed the comparison algorithm, it was not sufficient to demonstrate the superiority of the NCAAO algorithm. Due to the random nature of the metaheuristic, and for a fair comparison, the Wilcoxon rank sum test statistical analysis was used to draw statistically significant conclusions [43]. Simulation results for testing the proposed NCAAO using five competing algorithms are presented in Table 8. In this table, when the *p*-value is less than 0.05, the proposed NCAAO has a significant advantage over the competing algorithms in this group of objective functions. The *p*-values in the table also prove that the advantage is significant in most cases. This proves the high exploitation capability of the proposed algorithm, which is due to a proposed adaptive adjustment strategy of de-searching preferences that allows the algorithm to balance effectively in development and exploration. The effective combination of small habitat techniques allows the algorithm to speed up the process of preference seeking. In addition, the *p*-value also shows that the NCAAO algorithm effectively avoids the zero-search preference problem, proving that the results of the algorithm are very competitive.

**Figure 6:**
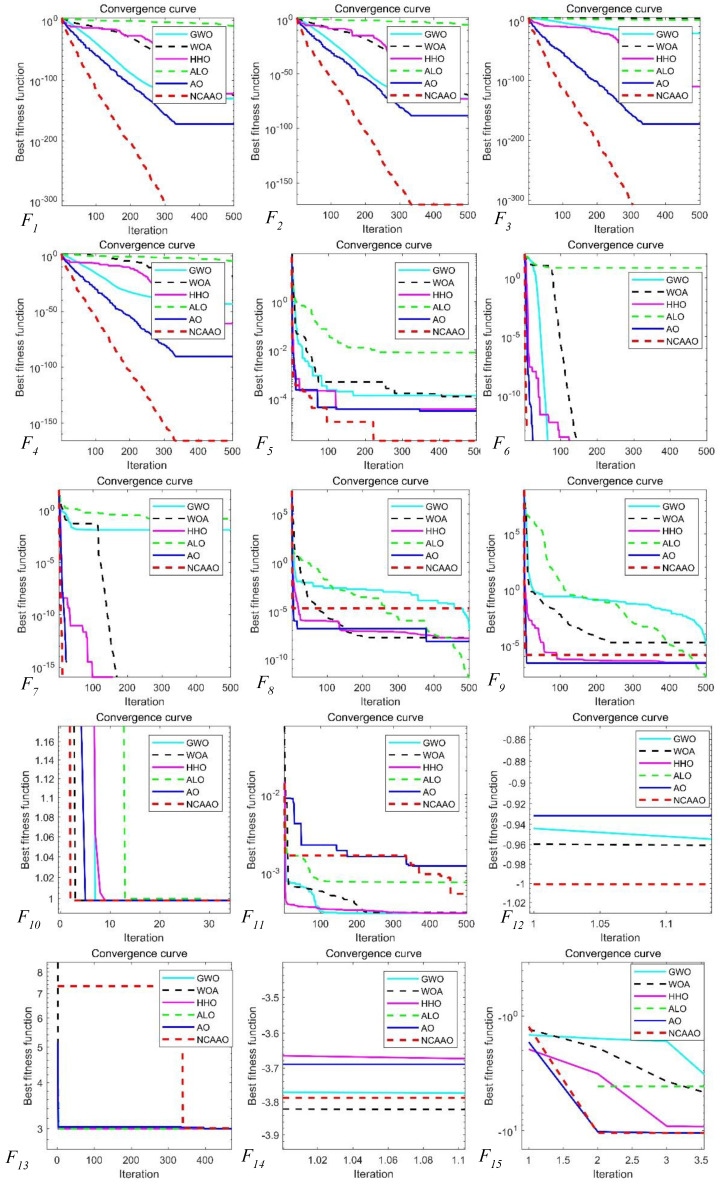
Convergence comparison curves of experiment.

To evaluate the convergence performance of the NCAAO algorithm, the convergence curves were plotted by selecting the best average value among 30 iterations, as shown in Figure 6. The convergence curves for the unimodal functions, high-dimensional multimodal functions and fixed-dimensional multimodal functions are shown in the figure. The curves show that for the single-peaked and multi-peaked functions, the NCAAO algorithm has a better ability to find the best and converges faster, which further proves that the algorithm has a better ability to develop as well as explore. As for the multimodal functions, the NCAAO algorithm fluctuates more during the initial iterations and relatively less so in the later stages. The significant decrease of the curve during the iterative process shows that the proposed communication exchange strategy based on the idea of small habitats increases the ability to update the optimal adaptation of the population. The overall iterative curve analysis concludes that the NCAAO algorithm has a stronger convergence behavior relative to the comparison algorithm. It is further demonstrated that NCAAO has a better ability to balance exploitation and exploration.

In summary, the NCAAO algorithm has significantly superior performance compared to other more classical optimization algorithms (e.g., GWO, WOA, HHO, ALO, AO). From the data in Table 5 and Table 6, we can see that the performance of the optimization search is better on the unimodal functions as well as the high-dimensional multimodal functions, and there is no significant decrease with the increase of the dimensionality. At the same time, through the convergence analysis in Figure 6, the NCAAO algorithm has good convergence overall; although the convergence speed is slower in function F8 compared with other functions, it can still find the optimal solution. It can be seen through the fixed-dimensional multimodal function’s test data in Table 7 that the optimal solution can still be found accurately when the optimal solution is not zero, which reduces the existence of zero search preference. Compared with other algorithms, the NCAAO algorithm has a strong balance of development ability and search ability.

### 4.2. Engineering Benchmark Sets

In order to further demonstrate that the NCAAO algorithm has better performance in finding the optimum, and to prove the application value of the algorithm in solving engineering problems, the tension/compression spring design problem [44], the pressure vessel design problem [45] and the three-bar truss design problem [46] were selected for solution testing. Salp Swarm Algorithm (SSA) [47], Whale Optimization Algorithm (WOA) [39], Gray Wolf Optimization (GWO) [38], Moth-flame Optimization MFO [48], Gravitational Search Algorithm GSA [49], Particle Swarm Optimization (PSO) [9], Genetic Algorithm (GA) [10], Tunicate Swarm Algorithm (TSA) [50] and NCAAO were also selected for comparison with the NCAAO algorithm for optimization testing of engineering examples. The values of the control parameters for these algorithms are likewise described in Table 4.

#### 4.2.1. Tension/Compression Spring Design Problem

**Figure 7:**
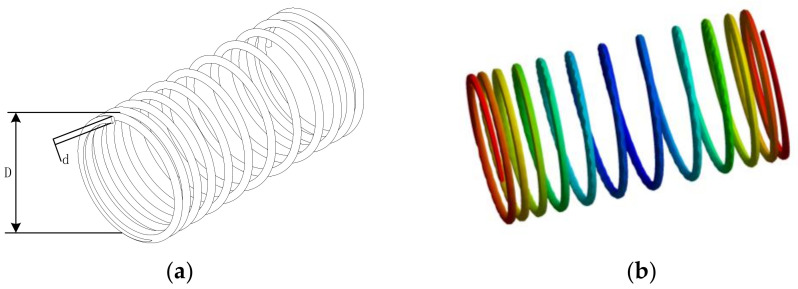
Schematic diagram of a tension/compression spring. (**a**) Schematic of the spring, (**b**) stress distribution evaluated at the optimum design.

The purpose of the tension/compression spring design problem is to minimize the weight of the pull-pressure spring, which is shown schematically in Figure 7. The main considerations in the design process are the cross-sectional diameter of the spring (d), the average coil diameter (D) and the number of active coils (Q).

The mathematical model of this problem is as follows:

Consider

(38) $$x=\left[x_{1},x_{2},x_{3}\right]=\left[d,D,Q\right]$$

Minimize

(39) $$f\left(x\right)=(x_{3}+2)\cdot x_{2}\cdot x_{1}^{2}$$

Subject to

(40) $$\left\{\begin{array}{c} g_{1}(X)=1-\frac{x_{2}^{3}\cdot x_{3}}{717854x_{1}^{4}}\leq 0\\ g_{2}(X)=\frac{4x_{2}^{2}-x_{1}x_{2}}{12566(x_{2}x_{1}^{3}-x_{1}^{4})}\leq 0\\ g_{3}(X)=1-\frac{140.45x_{1}}{x_{2}^{2}\cdot x_{3}}\leq 0\\ g_{4}(X)=\frac{x_{1}+x_{2}}{1.5}-1\leq 0 \end{array}\right.$$

Variable range

$$\left\{\begin{array}{l} 0.05\leq x_{1}\leq 2.00\\ 0.25\leq x_{2}\leq 1.30\\ 2.00\leq x_{3}\leq 15.00 \end{array}\right.$$

The NCAAO is applied to this case based on 30 independent runs with 500 group individuals and 500 iterations in each run. Since this benchmark case has some constraints, we need to integrate the NCAAO with a constraint handling technique. The results of NCAAO are compared to those reported for eight algorithms in the previous literature. Table 9 shows the detailed results of the proposed NCAAO compared to other techniques. Based on the results in Table 9, it is observed that NCAAO can reveal very competitive results compared to the MFO algorithm. Additionally, the NCAAO outperforms other optimizers significantly. The results obtained show that the NCAAO is capable of dealing with a constrained space.

#### 4.2.2. Pressure Vessel Design Problem

**Figure 8:**
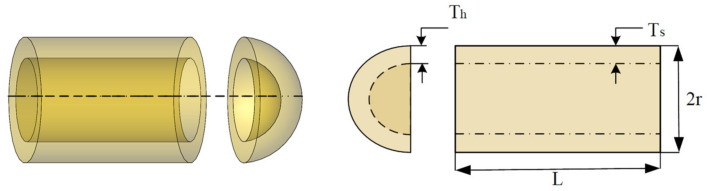
Pressure vessel design problem.

The pressure vessel design problem is a minimization problem, which is shown schematically in Figure 8. The variables of this case are thickness of shell (*Ts*), thickness of the head (*T_h_*), inner radius (*r*) and length of the section without the head (*L*).

The formulation of this test case is as follows:

Consider

(41) $$x=\left[x_{1},x_{2},x_{3},x_{4}\right]=\left[T_{s},T_{h},R,L\right]$$

Minimize

(42) $$f\left(x\right)=0.6224x_{1}x_{2}x_{3}+1.778x_{2}x_{3}^{2}+3.1661x_{1}^{2}x_{4}+19.84x_{1}^{2}x_{3}$$

Subject to

(43) $$\left\{\begin{array}{c} g_{1}(x)=-x_{1}+0.0193x_{3}\leq 0\\ g_{2}(x)=-x_{2}+0.00954x_{3}\leq 0\\ g_{3}(x)=-\pi x_{3}^{2}x_{4}-\frac{4}{3}\pi x_{3}^{3}+1296000\leq 0\\ g_{4}(x)=x_{4}-240\leq 0 \end{array}\right.$$

Variable range

$$\left\{\begin{array}{l} 0\leq x_{1},x_{2}\leq 100\\ 10\leq x_{3},x_{4}\leq 200 \end{array}\right.$$

Table 10 gives the optimal design results for the pressure vessels. The data in the table shows that NCAAO has given the relatively best results compared with other algorithms. Therefore, NCAAO is proven to be the best optimizer to deal with this problem in this test.

#### 4.2.3. Three-Bar Truss Design Problem

**Figure 9:**
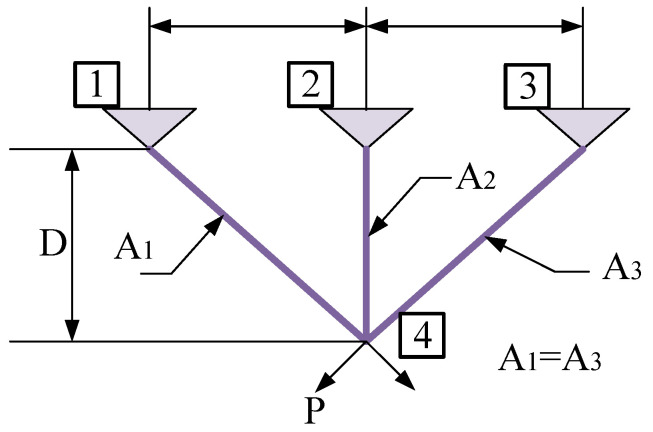
Three-bar truss design problem.

The three-bar truss design problem is a more classical problem that is used to test the performance of numerous algorithms. The schematic diagram of its structure and the relationship between the forces of each part are shown in Figure 9. The mathematical model of the problem is as follows:

Consider

(44) $$x=\left[x_{1},x_{2}\right]=\left[A_{1},A_{2}\right]$$

Minimize

(45) $$f\left(x\right)=(2\sqrt{2}x_{1}+x_{2})\times 1$$

Subject to

(46) $$\left\{\begin{array}{c} g_{1}(x)=\frac{\sqrt{2}x_{1}+x_{2}}{\sqrt{2}x_{1}^{2}+2x_{1}x_{2}}P-\sigma \leq 0,\\ g_{2}(x)=\frac{x_{2}}{\sqrt{2}x_{1}^{2}+2x_{1}x_{2}}P-\sigma \leq 0,\\ g_{3}(x)=\frac{1}{\sqrt{2}x_{2}+2x_{1}x_{2}}P-\sigma \leq 0, \end{array}\right.$$

Variable range

$$0\leq x_{1},x_{2}\leq 1$$

where, l = 100 cm, P = 2 KN/cm^2^, σ = 2 KN/cm^2^.

Table 11 reports the optimum designs attained by NCAAO and the listed optimizers. Inspecting the results in Table 11, we detected that the NCAAO is the best optimizer in dealing with problems and can attain superior results compared to other techniques.

## 5. Conclusions and Prospect

In this work, a new meta-heuristic algorithm called NCAAO is proposed for the AO algorithm, which is prone to fall into local optima and has the property of zero-point search preference and slow convergence. In fact, three new modules are integrated into the AO algorithm. The first one is the DLCS chaotic mapping chosen as the initial Skyhawk population generator. The resulting population has a more uniform distribution of individuals, which further clarifies the convergence direction of the algorithm effectively. The second module goes to the adaptive adjustment strategy of the search preferences, which is used to balance global search and local probing by changing the threshold of the Aquila selection work mechanism. Then, the adaptive position weight parameter is introduced to give a perturbation to the group individuals, which in turn updates their positions and improves the algorithm’s local exploitation capability. Finally, in the third module we propose a communication exchange strategy based on the idea of small habitats to better select the global optimum through the information exchange between individuals and to promote the rapid convergence of population individuals toward the global optimum and reduce the search error. Although chaotic mappings have been used in other algorithms, this work took into consideration the homogeneity of chaotic mappings to produce populations. Similarly, the existence of zero search preferences in the AO algorithm was investigated and the new algorithm was further improved.

The following improvements from the theoretical aspect demonstrate that the NCAAO algorithm shows excellent performance in the unified combination of theory and practice:

- Chaotic mapping has the characteristics of chaotic characteristics, randomness, ergodicity and regularity; the use of DLCS mapping produces a more uniform population distribution, which is conducive to an efficient search of the overall search space and further clarifies the convergence direction of the algorithm.
- Changing the threshold *adaptive_p* of the Aquila selection work and introducing the dynamic adjustment strategy in the development and exploration process help the algorithm to choose a reasonable strategy for the search area to seek the best and enhance the utilization behavior of NCAAO in the iterative process.
- The introduced adaptive location weight parameter ζ, which makes the group individual locations more diverse, further promotes the exploration behavior of NCAAO in the iterative process and has a constructive impact on balancing the exploitation and exploration trends.
- The proposed communication exchange strategy based on the idea of small habitats better promotes the optimization of group adaptation. Among them, a penalty mechanism is given to individuals with lower fitness to promote the NCAAO algorithm to better solve the problems of difficult high-dimensional search, local optimal incentive and unclear convergence direction.

The experiments further investigated the development performance, search performance, and local optimum avoidance performance of NCAAO by fifteen objective functions. The results show that NCAAO has good optimization performance. Meanwhile, three engineering application examples further verify that the NCAAO algorithm provides a better solution for practical engineering applications. Statistical analysis by the Wilcoxon rank sum test concludes that NCAAO has statistical significance. Based on the comprehensive study, it can be concluded that the proposed algorithm has significant advantages in solving practical problems and thus can provide different areas for other researchers. The results demonstrate that the NCAAO algorithm has a more intelligent balancing mechanism in the development and exploration process, which greatly circumvents the local optimal solutions and converges quickly toward the optimal solution when solving many different types of problems. At the same time, the existence of search preferences is suppressed, and the ability to jump out of the local optimal solution is significantly improved.

In future research, we plan to investigate the application of the proposed algorithm to problems such as image enhancement, feature extraction and camera calibration. In addition, we will further investigate in depth the effect of introducing chaotic mappings to evolve the initial population on the convergence performance, which is essential to enhance the performance of the algorithm.

## Figures and Tables

**Table 1:** Standard AO optimization process performance splitting.

Test Module	F_1__fit	F_10__fit	F_13__fit
Equation (3)	2.21 × 10^−160^	8.4767	3.3478
Equation (5)	86.42499	0.996	3.2516
Equation (12)	1.53 × 10^−4^	12.6705	5.3837
Equation (13)	0.0018084	1.998	3.0893

**Table 2:** Planar uniformity comparison.

U	Logistic	Cubic	Tent	DLCS
U_max_	1.4215	1.3726	1.0259	1.0238
U_min_	1.3201	1.1836	0.9931	0.9825
U_avg_	1.3698	1.2145	1.0241	1.0026

**Table 3:** Unimodal benchmark functions.

Function	Dim	Range	f_min_
$F_{1}\left(x\right)=\sum_{i=1}^{n}x_{i}^{2}$	10,30,100,500	[−100, 100]	0
$F_{2}\left(x\right)=\sum_{i=1}^{n}\left|x_{i}\right|+\prod_{i=1}^{n}\left|x_{i}\right|$	10,30,100,500	[−10, 10]	0
$F_{3}\left(x\right)={\sum_{i=1}^{n}\left(\sum_{j-1}^{i}x_{j}\right)}^{2}$	10,30,100,500	[−100, 100]	0
$F_{4}\left(x\right){=\max}_{i}\{\left|x_{i}\right|,1\leq i\leq n\}$	10,30,100,500	[−100, 100]	0
$F_{5}\left(x\right)=\sum_{i=1}^{n}ix_{i}^{4}+random[0,1)$	10,30,100,500	[−1.28, 1.28]	0
$F_{6}=\sum_{i=1}^{n}\left[x_{i}^{2}-10\cos\left(2\pi x_{i}\right)+10\right]$	10,30,100,500	[−5.12, 5.12]	0
$F_{7}(x)=\frac{1}{4000}\sum_{i=1}^{n}x_{i}^{2}-\prod_{i=1}^{n}\cos(\frac{x_{i}}{\sqrt{i}})+1$	10,30,100,500	[−600, 600]	0
$\begin{array}{l} F_{8}=\frac{\pi }{n}\{10\sin(\pi y_{1})\}+\\ \sum {}_{i=1}^{n-1}{\left(y_{i}-1\right)}^{2}[1+10\sin^{2}\left(\pi y_{i+1}\right)+\\ \sum {}_{i=1}^{n}u(x_{i},10,100,4)],where\begin{array}{cc} & y_{i}= \end{array}\\ 1+\frac{x_{i}+1}{4},u(x_{i},a,k,m)\left\{\begin{array}{c} K{\left(x_{i}-a\right)}^{m},x_{i}>a\\ 0,-a\leq x_{i}\leq a\\ K{\left(-x_{i}-a\right)}^{m},x_{i}<-a \end{array}\right. \end{array}$	10,30,100,500	[−50, 50]	0
$\begin{array}{l} F_{9}(x)=0.1\{\sin^{2}(3\pi x_{1})+{\sum_{i=1}^{n}\left(x_{i}-1\right)}^{2}\\ \times [1+\sin^{2}(3\pi x_{i}+1)]+{\left(x_{n}-1\right)}^{2}\left[1+\sin^{2}(2\pi x_{n})\right]\}\\ +\sum_{i=1}^{n}u\left(x_{i},5,100,4\right) \end{array}$	10,30,100,500	[−50, 50]	0
$F_{10}={\left(\frac{1}{500}+\sum {}_{j=1}^{25}\frac{1}{j+\sum {}_{i=1}^{2}}(x_{i}-a_{ij})\right)}^{-1}$	2	[−65, 65]	1
$F_{11}\left(x\right)={\sum_{i=1}^{11}\left[a_{i}-\frac{x_{1}\left(b_{i}^{2}+b_{i}x_{2}\right)}{b_{i}^{2}+b_{i}x_{3}+x_{4}}\right]}^{2}$	4	[−5, 5]	0.00030
$F_{12}=4x_{1}^{2}-2.1x_{1}^{4}+\frac{1}{3}x_{1}^{6}+x_{1}x_{2}-4x_{2}^{2}+4x_{2}^{4}$	2	[−5, 5]	−1.0316
$\begin{array}{l} F_{13}(x)=[1+{\left(x_{1}+x_{2}+1\right)}^{2}(19-14x_{1}+3x_{1}^{2}\\ -14x_{2}+6x_{1}x_{2}+3x_{2}^{2})]\times [30+{\left(2x_{1}-3x_{2}\right)}^{2}\\ \times (18-32x_{1}+12x_{1}^{2}-48x_{2}+36x_{1}x_{2}+27x_{2}^{2})] \end{array}$	2	[−2, 2]	3
$F_{14}=-\sum_{i=1}^{4}c_{i}\exp(-\sum_{i=1}^{3}a_{ij}{\left(x_{j}-p_{ij}\right)}^{2})$	3	[−1, 2]	−3.86
$F_{15}\left(x\right)=-{\sum_{i=1}^{10}[(X-a_{i}){\left(X-a_{i}\right)}^{T}+c_{i}]}^{-1}$	4	[0, 1]	−10.5363

**Table 4:** Parameter settings.

Algorithm	Setting
GWO	a was linearly decreased from 2 to 0, rate = 3
WOA	α decreased from 2 to 0, b = 1
HHO	t = 0
ALO	*I* ratio = 10^ω^, ω = [2, 6]
AO	S = 1.5, r_1_ take a fixed index between 1 and 20, G_2_ decreased from 2 to 0
MFO	Convergence constant α from 2 to 0, Spiral factor b = 1
PSO	C_1_ = −0.2, C_2_ = 1.49445, w = 0.8 P_c_ = 0.95,
GA	P_c_ = 0.95, P_m_ = 0.001, E_r_ = 0.2
TSA	t = 0
SSA	P_percent = 0.2

**Table 5:** The comparison of obtained solutions for unimodal functions.

F	D	Index	GWO	WOA	HHO	ALO	AO	NCAAO
F1	10	AVG	5.6575 × 10^−131^	4.8691 × 10^−122^	9.5173 × 10 ^−122^	3.2913 × 10^−10^	5.6144 × 10^−174^	**0**
		STD	1.4541 × 10^−130^	1.0843 × 10^−121^	2.6643 × 10^−121^	1.1628 × 10^−10^	0	0
	30	AVG	5.4833 × 10^−57^	1.3682 × 10^−115^	5.8876 × 10^−118^	4.8172 × 10^−8^	4.2005 × 10^−176^	**0**
		STD	1.3376 × 10^−56^	2.0943 × 10^−115^	1.2566 × 10^−117^	2.0373 × 10^−8^	0	0
	100	AVG	1.1543 × 10^−25^	2.6843 × 10^−114^	3.2317 × 10^−115^	3.8028 × 10^−2^	1.381 × 10^−177^	**0**
		STD	2.5685 × 10^−25^	3.8666 × 10^−113^	5.6943 × 10^−114^	2.6591 × 10^−2^	0	0
F2	10	AVG	7.7136 × 10^−72^	1.0135 × 10^−67^	3.6286 × 10^−67^	1.9342 × 10^−2^	5.2349 × 10^−87^	**5.8837 × 10^−169^**
		STD	1.4544 × 10^−71^	1.60484 × 10^−67^	7.06325 × 10^−67^	4.3151 × 10^−2^	1.1556 × 10^−86^	0
	30	AVG	3.3720 × 10^−33^	4.99 × 10^−67^	4.3415 × 10^−69^	2.2323	1.0200 × 10^−88^	**1.9000 × 10^−168^**
		STD	3.94207 × 10^−33^	9.9288 × 10^−67^	4.30699 × 10^−67^	1.5552	1.86559 × 10^−88^	0
	100	AVG	2.7785 × 10^−14^	1.2822 × 10^−63^	4.1000 × 10^−68^	8.0225 × 10^2^	1.4752 × 10^−88^	**5.8922 × 10^−167^**
		STD	3.5943 × 10^−14^	2.4739 × 10^−63^	3.5430 × 10^−67^	1.1591 × 10^2^	1.7100 × 10^−88^	0
F3	10	AVG	6.7187 × 10^−76^	4.3830 × 10^−6^	1.4170 × 10^−106^	1.6350 × 10^−6^	1.4380 × 10^−178^	**0**
		STD	2.3663 × 10^−75^	1.36690 × 10^−6^	0	7.3425 × 10^−6^	0	0
	30	AVG	4.1523 × 10^−22^	4.2241 × 10^−3^	4.4823 × 10^−111^	2.1156 × 10^1^	2.3572 × 10^−173^	**0**
		STD	1.2303 × 10^−22^	5.0500 × 10^−3^	0	7.6463 × 10^1^	0	0
	100	AVG	1.4000 × 10^−3^	4.1766 × 10^5^	1.8609 × 10^−107^	1.2330 × 10^4^	1.3141 × 10^−177^	**0**
		STD	5.2473 × 10^−2^	2.2046 × 10^5^	5.5540 × 10^−107^	8.1439 × 10^4^	0	0
F4	10	AVG	2.5343 × 10^−44^	1.1055 × 10^−8^	6.3666 × 10^−60^	1.5402 × 10^−5^	1.9843 × 10^−89^	**7.9443 × 10^−167^**
		STD	3.4655 × 10^−44^	2.4773 × 10^−8^	7.6522 × 10^−60^	3.0500 × 10^−6^	2.5100 × 10^−89^	0
	30	AVG	8.1471 × 10^−15^	4.4538 × 10^−14^	4.4883 × 10^−59^	3.4741	1.1948 × 10^−86^	**2.0741 × 10^−167^**
		STD	7.7544 × 10^−15^	8.5664 × 10^−14^	9.9273 × 10^−59^	3.7564	2.6700 × 10^−86^	0
	100	AVG	4.4000 × 10^−5^	1.7645 × 10^−1^	7.6788 × 10^−60^	2.0600 × 10^1^	1.1767 × 10^−86^	**5.0800 × 10^−168^**
		STD	1.5946 × 10^−5^	3.4955 × 10^−1^	8.6634 × 10^−60^	2.5973	2.3443 × 10^−86^	0
F5	10	AVG	2.7156 × 10^−4^	2.0000 × 10^−2^	1.6343 × 10^−5^	3.0100 × 10^−3^	9.2146 × 10^−5^	**4.3222 × 10^−12^**
		STD	3.0725 × 10^−4^	3.5740 × 10^−2^	2.0440 × 10^−5^	3.3140 × 10^−3^	1.3600 × 10^−4^	0
	30	AVG	6.8043 × 10^−5^	2.6225 × 10^−3^	1.7400 × 10^−4^	3.8846 × 10^−3^	1.5622 × 10^−4^	**2.0510 × 10^−6^**
		STD	2.0579 × 10^−5^	2.8950 × 10^−3^	3.2803 × 10^−4^	1.9460 × 10^−3^	2.0864 × 10^−4^	0
	100	AVG	2.2536 × 10^−4^	1.9441 × 10^−4^	1.8113 × 10^−5^	3.7800 × 10^−1^	1.8843 × 10^−4^	**5.2014 × 10^−6^**
		STD	1.8141 × 10^−4^	3.3940 × 10^−4^	1.8235 × 10^−5^	3.1397 × 10^−1^	3.8878 × 10^−4^	1.5420 × 10^−6^
RANK	10	w/t/l	0/0/5	0/0/5	0/0/5	0/0/5	0/0/5	5/0/0
	30	w/t/l	0/0/5	0/0/5	0/0/5	0/0/5	0/0/5	5/0/0
	100	w/t/l	0/0/5	0/0/5	0/0/5	0/0/5	0/0/5	5/0/0

**Table 6:** The comparison of obtained solutions for multimodal functions.

F	D	Index	GWO	WOA	HHO	ALO	AO	NCAAO
F6	10	AVG	4.2526	2.9536 × 10^−15^	**0**	1.7724 × 10^1^	**0**	**0**
		STD	5.9828	6.8724 × 10^−15^	0	1.1325 × 10^1^	0	0
	30	AVG	2.1300 × 10^1^	**0**	**0**	3.7500 × 10^2^	**0**	**0**
		STD	1.3500 × 10^1^	0	0	6.8300 × 10^1^	0	0
	100	AVG	1.4028 × 10^1^	**0**	**0**	3.3645 × 10^2^	**0**	**0**
		STD	4.1562	0	0	4.0162 × 10^1^	0	0
F7	10	AVG	4.9425 × 10^−3^	4.4737 × 10^−2^	**0**	1.7114 × 10^−1^	**0**	**0**
		STD	6.7643 × 10^−3^	6.2962 × 10^−2^	0	1.0108 × 10^−1^	0	0
	30	AVG	4.1982 × 10^−2^	**0**	**0**	2.2600 × 10^−2^	**0**	**0**
		STD	3.6423 × 10^−2^	0	0	2.4700 × 10^−1^	0	0
	100	AVG	2.7342 × 10^−2^	1.3320 × 10^−1^	**0**	2.3934 × 10^−1^	**0**	**0**
		STD	2.2163 × 10^−2^	1.5432 × 10^−1^	0	1.5643 × 10^−1^	0	0
F8	10	AVG	2.0436 × 10^−2^	1.1674 × 10^−2^	1.4803 × 10^−4^	2.8749 × 10^−2^	3.1236 × 10^−4^	**1.8510 × 10^−5^**
		STD	8.8876 × 10^−3^	8.9764 × 10^−2^	5.2234 × 10^−4^	1.2356 × 10^−3^	4.2360 × 10^−5^	3.2664 × 10^−5^
	30	AVG	6.1270 × 10^−1^	4.7881 × 10^−1^	2.0856 × 10^−6^	1.5716 × 10^1^	1.0767 × 10^−6^	**1.2786 × 10^−7^**
		STD	1.5289 × 10^−1^	2.3845 × 10^−1^	1.1722 × 10^−5^	2.1337 × 10^1^	1.4585 × 10^−6^	3.2361 × 10^−7^
	100	AVG	5.2230 × 10^−2^	1.3180 × 10^−1^	4.2344 × 10^−5^	2.4432 × 10^1^	7.3494 × 10^−6^	**6.7628 × 10^−6^**
		STD	1.3462 × 10^−2^	1.5785 × 10^−1^	5.4672 × 10^−5^	1.3258 × 10^1^	8.7032 × 10^−6^	1.3640 × 10^−6^
F9	10	AVG	4.0664 × 10^−2^	7.3654 × 10^−2^	1.5774 × 10^−4^	5.0367 × 10^−3^	9.4682 × 10^−5^	**5.4800 × 10^−7^**
		STD	9.0056 × 10^−2^	6.3332 × 10^−2^	2.5761 × 10^−4^	5.9886 × 10^−3^	0.3021 × 10^−2^	2.6473 × 10^−6^
	30	AVG	4.0500	3.7500	1.5736 × 10^−4^	9.6700 × 10^1^	6.4589 × 10^−5^	**1.3468 × 10^−6^**
		STD	4.8600	5.4500 × 10^−1^	1.8932 × 10^−4^	7.5600 × 10^1^	6.6508 × 10^−5^	5.6430 × 10^−6^
	100	AVG	3.6753 × 10^−1^	4.3929 × 10^−1^	9.1331 × 10^−4^	1.3463 × 10^1^	1.8243 × 10^−5^	**2.8756 × 10^−4^**
		STD	1.0467 × 10^−1^	3.1817 × 10^−1^	1.2332 × 10^−3^	1.2789 × 10^1^	1.6473 × 10^−5^	7.6312 × 10^−4^
RANK	10	w/t/l	0/0/4	0/0/4	0/2/2	0/0/4	0/2/2	2/2/0
	30	w/t/l	0/0/4	0/1/3	0/2/2	0/0/4	0/2/2	2/2/0
	100	w/t/l	0/0/4	0/0/4	0/2/0	0/0/4	0/2/2	2/2/0

**Table 7:** The comparison of obtained solutions for fixed-dimension multimodal functions.

F	D	Index	GWO	WOA	HHO	ALO	AO	NCAAO
F10	2	AVG	**9.9800 × 10^−1^**	**9.9800 × 10^−1^**	**9.9800 × 10^−1^**	**9.9800 × 10^−1^**	**9.9800 × 10^−1^**	**9.9800 × 10^−1^**
		STD	0	0	0	0	0	0
F11	4	AVG	3.1666 × 10^−4^	3.2614 × 10^−4^	**3.1002 × 10^−4^**	5.6676 × 10^−4^	5.1303 × 10^−4^	5.4825 × 10^−4^
		STD	1.9293 × 10^−5^	3.5372 × 10^−5^	3.3390 × 10^−6^	2.2070 × 10^−4^	4.0083 × 10^−4^	2.0710 × 10^−6^
F12	2	AVG	**−1.0316**	**−1.0316**	**−1.0316**	**−1.0316**	**−1.0316**	**−1.0316**
		STD	0	0	0	0	0	0
F13	2	AVG	**3**	**3**	**3**	**3**	**3**	3.0053
		STD	0	0	0	0	0	1.3000 × 10^−4^
F14	3	AVG	−3.8623	−3.8596	−3.8347	−3.8213	−3.8425	**−3.8604**
		STD	1.6897 × 10^−3^	2.1255 × 10^−2^	4.0300 × 10^−2^	0.4283 × 10^−2^	0.1978 × 10^−2^	7.7618 × 10^−4^
F15	4	AVG	−1.0543 × 10^1^	−2.1105 × 10^4^	−7.2953	−7.2943	−2.1134 × 10^4^	**−1.0500 × 10^1^**
		STD	1.0832 × 10^−4^	4.8980 × 10^−5^	2.9620	2.9620	1.0496 × 10^−4^	7.4736 × 10^−4^
RANK	D	w/t/l	0/3/2	0/3/2	1/3/2	0/3/3	0/3/3	2/2/1

**Table 8:** The *p*-values obtained from Wilcoxon sum rank test. (*p ≥* 0.05 have been underlined).

Functions Type	GWO and NCAAO	WOA and NCAAO	HHO and NCAAO	ALO and NCAAO	AO and NCAAO
Unimodal	0.000639	0.000248	0.001553	0.000010	0.009578
High-dimensional multimodal	0.028571	0.065714	0.065714	0.028571	0.082857
Fixed-dimensional multimodal	0.011893	0.007800	0.003965	0.007800	0.068436

**Table 9:** Comparison of results for tension/compression spring problem.

Algorithm	Optimum Variables			Optimum Weight
	d	D	N	
SSA [47]	0.051197	0.345219	12.00402	0.0126821
WOA [39]	0.05119	0.357236	12.00309	0.0126828
GWO [38]	0.05171	0.354382	11.28698	0.0125433
MFO [48]	0.051843	0.364107	11.24036	0.0126734
GSA [49]	0.05019	0.353697	14.25410	0.126963
PSO [9]	0.05031	0.310225	14.00324	0.013108
GA [10]	0.05034	0.315979	15.24316	0.012833
TSA [50]	0.050109	0.341599	12.07350	0.012655
NCAAO	0.051836	0.360026	11.13659	0.0126649

**Table 10:** Comparison of results for pressure vessel design problem.

Algorithm	Optimum Variables				Optimum Cost
	T_s_	T_h_	R	L	
SSA [47]	0.812500	0.437516	42.09517	176.9635	6059.7294
WOA [39]	0.783694	0.391106	40.60043	200	5923.497
GWO [38]	0.85257	0.421394	44.23575	175.5324	6142.5731
MFO [48]	0.81150	0.441350	42.10036	176.3492	6058.3024
GSA [49]	1.08996	0.956638	49.73246	183.6672	8766.9234
PSO [9]	0.75536	0.424861	41.65367	179.1068	5919.763
GA [10]	1.095612	0.920064	44.67365	182.5634	6587.639
TSA [50]	0781681	0.386526	40.31256	200	5919.263
NCAAO	0.817624	0.417563	42.98364	177.8329	6000.3776

**Table 11:** Comparison of results for tree-bar truss design problem.

Algorithm	Optimum Variables		Optimum Weight
	x_1_	x_2_	
SSA [47]	0.791462	0.407936	264.10593
WOA [39]	0.786629	0.406775	263.89534
GWO [38]	0.786513	0.408248	264.01956
MFO [48]	0.787236	0.406624	263.89511
GSA [49]	0.790254	0.407993	263.97544
PSO [9]	0.788522	0.408346	263.89602
GA [10]	0.794366	0.395462	264.00395
TSA [50]	0.789560	0.408011	262.97637
NCAAO	0.788653	0.408294	263.89582

## Data Availability

The data presented in this study are available on request from the corresponding author. The data are not publicly available due to Project Confidential.
